# Clinical communication in orthodontics: Any questions?

**DOI:** 10.1177/14653125221084314

**Published:** 2022-03-18

**Authors:** Daniel Stonehouse-Smith, Nikolaos Pandis, Dirk Bister, Jadbinder Seehra

**Affiliations:** 1 Department of Orthodontics, Faculty of Dentistry, Oral & Craniofacial Sciences, King’s College London, London, UK; 2 Department of Orthodontics and Dentofacial Orthopedics, Dental School/Medical Faculty, University of Bern, Bern, Switzerland

**Keywords:** psychological aspects of orthodontics, health services and quality of life aspects, risk/benefit, assessment

## Abstract

**Objective:**

To measure patient-perceived standards of clinician communication and identify elements of deficient performance. Good communication can improve the quality of care, patient satisfaction and compliance with treatment.

**Design:**

Cross-sectional questionnaire service evaluation.

**Setting:**

Two university dental hospital orthodontic departments.

**Participants:**

Any patients aged 10 years and over attending the orthodontic department for treatment or consultation were eligible for inclusion. Patients who required third-party translation services were excluded.

**Methods:**

Clinicians provided the modified 15-item Communication Assessment Tool (CAT) to up to five patients in a clinical session. A front sheet for clinician characteristics was used and anonymised with a unique identifier. Univariable logistic GEE models examined associations among responses and clinician characteristics.

**Results:**

There were 55 clinicians with 204 patient responses. The overall percentage of ‘5=excellent’ ratings was 88% (SD 0.16). The lowest scoring item was ‘encouraged me to ask questions’ (55.8%). Based on clinician characteristics, there were lower odds of an excellent response for certain CAT items. There were higher odds of an excellent response if English was not the clinician’s first language (1.05; 95% confidence interval = 1.00–1.09; *P*=0.03).

**Conclusion:**

There is a high standard of patient–clinician communication in the hospital orthodontic setting. Key areas of communication that require attention include encouraging patients to ask questions, talking in terms they can understand, recognising their main concerns and involving them in the decision-making process. The results of this study can be used to inform communication skills training and be replicated in similar dental settings (primary and secondary care) as part of quality improvement.

## Introduction

There is evidence to support that good patient–clinician communication can improve the quality of care (Stewart et al., 2000), patient satisfaction (Yamalik, 2005) and compliance with treatment (DiMatteo et al., 2012). When communication is patient-centred, patients can also feel empowered to partake in shared decision making about their care (Barber, 2019) and patient values and preferences are a pillar of evidence-based practice. Conversely, poor communication is widely accepted as a contributing factor in many complaints (Krause et al., 2001; Waylen, 2017). Effective communication is a key standard of the General Dental Council (2013), and registered dental professionals must demonstrate and maintain competence in this skill as part of their ongoing practice. Within the context of orthodontic interventions, a high burden of compliance rests with the patient. Communication skills are therefore a domain of the Orthodontics curriculum, as set by the Joint Committee for Postgraduate Training in Dentistry (2010) and developed with the Royal Colleges.

Communication can be assessed in daily practice through workplace-based assessments, Objective Structured Clinical Examinations (OSCEs), peer observation and feedback from colleagues. This normally centres around defined observations, such as explaining treatment options, or the likely risks involved. Patient perceptions are not necessarily considered, and their input is required for a holistic assessment of a clinician’s interpersonal skills. Patient satisfaction questionnaires are already widely used as part of service evaluation in the secondary care setting and patient-reported experience measures (PREMs) are becoming an increasingly important part of evaluating quality and outcomes in healthcare (Ryan and Cunningham, 2018).

We report the findings of a multicentre service evaluation into patient–clinician communication within the secondary care orthodontic setting using the Communication Assessment Tool (CAT); this being a reliable and validated instrument for patient-reported assessment of clinician’s interpersonal and communication skills (Makoul et al., 2007). The aim of the present study was to measure the patient-perceived standard of orthodontic clinicians’ communication and identify elements of communication where performance is deficient. Clinician factors which influence patient perceptions of communication were also explored.

## Materials and methods

This study was classified as a service evaluation and registered with the clinical governance departments at both Guys and St Thomas NHS Foundation Trust (10930) and Kings College London NHS Foundation Trust (DENT051-20). Ethical approval was not required for this study as it was classified as a service evaluation. Participation in this cross-sectional evaluation was entirely voluntary. Participants were recruited from orthodontic new patient and treatment clinics at both secondary care sites. Treatment clinics were heterogenous in nature and would also have included treatment planning, review appointments and consenting procedures. Any patients aged 10 years and over attending the orthodontic department for treatment or consultation were eligible for inclusion. Patients who required third-party translation services were excluded. As this was a service evaluation using a form of patient satisfaction questionnaire, a convenience sample of 200 patients (100 patients recruited at each site) was deemed large enough by the investigators to gain a baseline level of patient–clinician communication skills across the orthodontic clinics, comparable to a previous sample using the CAT in the dental hospital setting (Waylen et al., 2015). Due to the nature of the evaluation, a formal power calculation was not deemed necessary.

## Data collection tool

The CAT instrument was developed to capture patient views on interpersonal skills soon after an inpatient or outpatient clinical encounter, rather than over a period of time. Initial field testing and focus group discussion during the development of the tool specifically amalgamated domains on giving information about tests or investigations, diagnoses and treatment into a single item based on individual patient expectations (gave me as much information as I wanted). Separating these domains was felt to be too narrow and not applicable to all clinical interactions. The CAT was specifically designed and tested to be applicable across settings and specialties and was also validated with a sample of patients with whom the majority (69.7%) had seen their clinician more than once before (Makoul et al., 2007).

**Figure 1.**
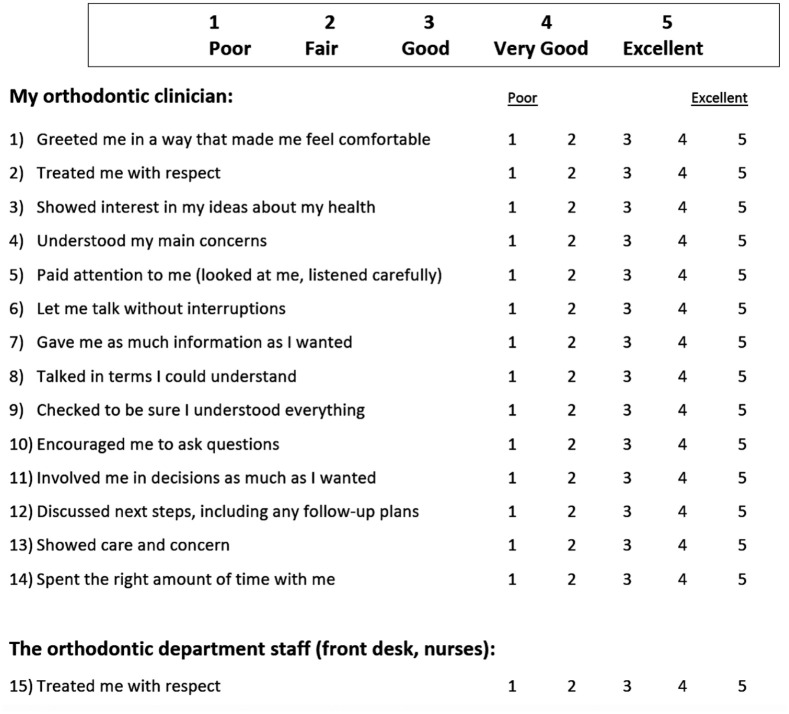
CAT questionnaire. Adapted from Makoul et al. (2007).

The CAT was adapted from the original tool reported by Makoul et al. (2007) on discussion with the local clinical governance team. The only changes were to the headers ‘The doctor’ and ‘The doctor’s staff’ to ‘Orthodontic clinician’ and ‘Orthodontic department staff (front desk, nurses)’ to apply it to the hospital orthodontic setting and clarify the individuals that patients were providing scores for (Figure 1). This is comparable to adaptations of the tool by Waylen et al. (2015) and Catt et al. (2018). The CAT comprises 15 statements such as ‘encouraged me to ask questions’, ‘gave me as much information as I wanted’ and ‘spent the right amount of time with me’. It takes only several minutes to complete (Makoul et al., 2007) and so is of minimal inconvenience to patients and has been piloted and validated across both medical and dental settings (Armellino et al., 2021; Catt et al., 2018; Ferranti et al., 2010; Makoul et al., 2007; Waylen et al., 2015). Each element is scored by patients on a 5-point Likert scale as follows: 1 = poor; 2 = fair; 3 = good; 4 = very good; and 5 = excellent. A mean score for the first 14 items is calculated for the individual clinician while the final item asks for feedback on other departmental staff, such as nurses or the front desk. Although only a snapshot of patient experience, it can provide an overall gauge of their perceptions of clinician’s communication skills. As most scores tend to be clustered towards the higher end of the scale, it is recommended to use the proportion of items rated ‘excellent’ as a more useful measure (Makoul et al., 2007). Psychometric evaluation has found that anything less than ‘excellent’ is better equated to this domain not being fully achieved in the eyes of the patient. The mean ‘excellent’ scores using the CAT can be calculated on the number of questions the patient answered, excluding those left blank, as suggested with previous use of this tool (Waylen et al., 2015).

The modified CAT is plain English with a Flesch Reading Ease score of 71.3 and a Flesch-Kincaid grade level of 5.4 and is therefore suitable for most US fifth graders (aged 10–11 years). This is a similar reading level to that reported in the literature for other versions of this tool (Ferranti et al., 2010; Makoul et al., 2007) and most patients aged 10 years and over should be able to understand the CAT, with some help from a guardian if needed. This age also conveniently includes most orthodontic patients. For younger individuals who asked for help from a guardian, their view was considered acceptable for completion of the CAT questionnaire. Any conflict was decided among the patient and their guardian away from the clinical area. To avoid confusion, patients were advised that ‘My orthodontic clinician’ refers to the primary treating clinician, those spending the most time with them during the appointment, and not that of a supervising consultant who may have provided a brief opinion.

Clinicians were given five paper copies of the CAT and asked to provide them to their patients at the start of a clinical session. Clinicians were not aware of the session being sampled beforehand and so were not able to modify bookings in advance. In addition to the questionnaire responses, the following demographics were collected: sex (patient and clinician); first language (patient and clinician); and grade and region of the primary registrable dental qualification of the clinician. Both clinician and patients were anonymised by providing each clinician with a unique identifier that was used on all questionnaires to enable data linkage. Patients were encouraged to complete the questionnaire at the end of the appointment, ideally away from the clinical area in the waiting room or a side room where available. A collection box was available in the reception area for patients to return their completed questionnaire. Data were collated and presented in aggregate using a pre-piloted Microsoft Excel (Microsoft, Redwood, WA, USA) spreadsheet. No patient identifiable information was recorded, and individualised feedback reports were also not provided to clinicians.

## Standards

Pilot data using the CAT for standards of clinical communication in the orthodontic setting have not yet been established. Previous studies have determined mean excellence scores that were used as a composite reference standard: 76.3% of scores were excellent in multiple medical settings (Makoul et al., 2007); 73.3% of scores were excellent in the Dental Hospital setting (Waylen et al., 2015); and 74.4% of scores were excellent in the orthognathic team setting (Catt et al., 2018). Based on these, a suggestive target standard was adopted where 75% of CAT scores should be rated excellent by patients. This is generally calculated as a mean percentage ‘excellent’ for the first 14 items of the CAT but can be broken down individually to highlight areas for improvement.

## Statistical analyses

Data analyses were carried out independently by a statistician (NP). Descriptive statistics and summary values were calculated. A series of univariable logistic Generalised Estimating Equation models were fit with empirical standard errors to examine associations among the response (excellent [response 5]) versus unsatisfactory [combined responses 1–4]) and the clinician characteristics. In the final model the significant predictors from the univariable analyses were included. A two-tailed *P* value of 0.05 was considered statistically significant. Statistical analyses were performed using STATA software version 16.1 (Stata Corp., College Station, TX, USA) and R Software version 4.0.3 (R Foundation for Statistical Computing, Vienna, Austria).

## Results

Data collection was commenced at both sites from 19 April 2021 and ended on 21 June 2021. This period was required to sample the desired population in the context of the COVID-19 pandemic and reduction in clinical capacity, as well as to account for the varying timetables of the clinicians involved. The characteristics of patients (n=204) and clinicians (n=55) involved in this service evaluation are presented in Table 1. The mean number of CAT questionnaires per clinician was 3.71 (SD 0.99) with the mean age of respondents being 17.9 (SD 7.9) years. In total, there were 275 CAT questionnaires provided to clinicians. Some patients may have failed to attend, failed to complete the CAT or simply fewer than five patients were booked in for that session. We did not collect unused CAT questionnaires, but the remaining 71 questionnaires may have been declined by patients or left blank for the aforementioned reasons. The final sample of 204 consisted of responses mainly from treatment clinics (n=181), with the remaining being from new patient consultations (n=23).

**Table 1.** Study characteristics (n=204).

Study characteristics	
*Clinician (n=55)*	
Hospital	
1. King’s College Hospital (KCH)	24 (43.6)
2. Guy’s Hospital (GSTT)	31 (56.4)
Clinician grade	
1. Trainee/Registrar	36 (65.5)
2. Therapist	7 (12.7)
3. Consultant/Staff	12 (21.8)
Gender	
1. Female	37 (67.3)
2. Male	18 (32.7)
Qualification region	
1. UK	34 (61.8)
2. EU	4 (7.3)
3. Other	17 (30.9)
English first language	
1. Yes	29 (52.7)
2. No	26 (47.3)
*Participants (n=204)*	
Hospital	
1. King’s College Hospital (KCH)	101 (49.5)
2. Guy’s Hospital (GSTT)	103 (50.5)
Sex	
1. Female	110 (53.9)
2. Male	92 (45.1)
3. Prefer not to say	2 (1.0)
English first language	
1. Yes	174 (85.3)
2. No	30 (14.7)
Age (years)	17.9 SD 7.9

Values are given as n (%) or mean (SD).

The overall responses and mean score per item are presented in Table 2 with no patients choosing to rate any of the domains as ‘1=poor’. The overall mean score for any given domain was 4.86 (SD 0.21). The percentage of ‘5=excellent’ ratings per domain are also shown with the overall percent-excellence for the first 14 items on the CAT being 88.2% (SD 0.16). The lowest scoring item was ‘encouraged me to ask questions’ with only 55.8% of responses indicating that this domain was fulfilled.

**Table 2.** Frequency of response scores, mean score per item and % excellent ratings per item.

	Response				Mean	Median	Ratings (% excellent)
CAT item	2 (fair)	3 (good)	4 (very good)	5 (excellent)			
1. Greeted me in a way that made me feel comfortable	0	3	9	192	4.93	5	94.1
2. Treated me with respect	0	0	8	196	4.96	5	96.0
3. Showed interest in my ideas about my health	1	2	11	190	4.91	5	93.1
4. Understood my main concerns	1	4	31	168	4.49	5	82.3
5. Paid attention to me (looked at me, listened carefully)	1	1	10	192	4.93	5	94.1
6. Let me talk without interruptions	1	0	9	194	4.94	5	95.1
7. Gave me as much information as I wanted	0	2	14	188	4.91	5	92.2
8. Talked in terms I could understand	1	2	40	161	4.77	5	78.9
9. Checked to be sure I understood everything	0	2	30	172	4.83	5	84.3
10. Encouraged me to ask questions	1	11	78	114	4.49	5	55.8
11. Involved me in decisions as much as I wanted	0	1	24	179	4.87	5	87.7
12. Involved me in decisions as much as I wanted	0	1	11	192	4.94	5	94.1
13. Showed care and concern	0	0	9	195	4.96	5	95.6
14. Spent the right amount of time with me	0	4	20	180	4.86	5	88.2
15. The orthodontic department staff treated me with respect	1	4	28	171	4.80	5	83.8

**Figure 2.**
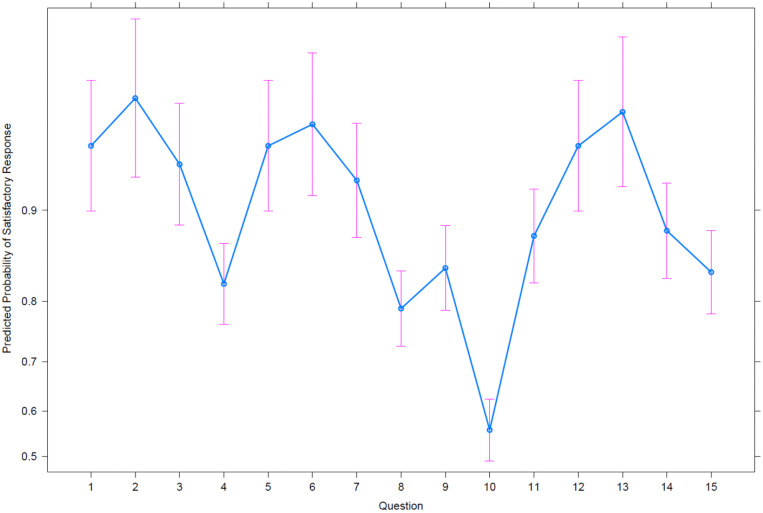
Predicted probability with 95% confidence intervals of an excellent versus unsatisfactory response (5 vs. 4–2) per question.

Predictors of excellent responses were also explored. Based on clinician characteristics, there were lower odds of achieving an excellent response for items 4, 8, 9, 10, 11 and 14 compared to item 1, as demonstrated in Table 3. A similar finding was made for item 15, although this domain is based on the orthodontic department staff rather than the individual clinician. There were higher odds of achieving an excellent response if English was not the first language of the treating clinician (1.05; 95% CI: 1.00,1.09; p=0.03). Other factors including clinician grade, gender, region of qualification and patient’s first language did not appear to alter the odds of achieving an excellent response. The predicted probabilities of achieving an excellent response for each of the CAT items from the GEE model are displayed in Figure 2.

**Table 3.** Estimates, 95% CIs and *P* values for the effect of clinician characteristics and type of question on the response excellent versus unsatisfactory (5 vs. 4–2).

		Univariable analysis		Multivariable analysis	
Predictor	Category	OR (95% CI)	*P* value	OR (95% CI)	*P* value
*Question (per unit)*					
	1	Reference			
	2	1.02 (0.96–1.07)	0.480	1.02 (0.97–1.08)	0.48
	3	0.99 (0.94–1.05)	0.724	0.99 (0.94–1.05)	0.72
	4	0.89 (0.84–0.94)	<0.001	0.89 (0.84–0.94)	<0.001
	5	1.00 (0.95–1.06)	1.000	1.00 (0.95–1.06)	1.00
	6	1.01 (0.96–1.07)	0.724	1.01 (0.96–1.07)	0.72
	7	0.98 (0.93–1.04)	0.480	0.98 (0.93–1.04)	0.48
	8	0.86 (0.81–0.91)	<0.001	0.86 (0.81–0.91)	<0.001
	9	0.91 (0.86–0.96)	<0.001	0.91 (0.86–0.96)	<0.001
	10	0.68 (0.65–0.72)	<0.001	0.68 (0.65–0.72)	<0.001
	11	0.94 (0.89–0.99)	0.02	0.94 (0.89–0.99)	0.02
	12	1.00 (0.94–1.06)	1.000	1.00 (0.95–1.06)	1.00
	13	1.02 (0.96–1.07)	0.596	1.02 (0.96–1.07)	0.60
	14	0.94 (0.89–0.99)	0.03	0.94 (0.89–0.99)	0.03
	15	0.90 (0.85–0.95)	<0.001	0.90 (0.85–0.95)	<0.001
*Hospital (per unit)*					
	KCH	Reference			
	GSTT	0.99 (0.95–1.03)	0.668		
*Clinician grade (per unit)*		1.05 (0.97–1.13)	1.05		
	Trainee	Reference			
	Therapist	1.05 (0.98–1.12)	0.125		
	Consultant	0.99 (0.94–1.04)	0.651		
*Sex (per unit)*					
	Female	Reference			
	Male	0.99 (0.95–1.04)	0.654		
*Qualification region (per unit)*					
	UK	Reference			
	EU	1.03 (0.95–1.12)	0.506		
	Other	1.03 (0.98–1.08)	0.224		
*English first language (per unit)*					
	Yes	Reference			
	No	1.05 (1.00–1.09)	0.03	1.05 (1.00–1.09)	0.03*

## Discussion

The results of this service evaluation show that there was a high standard of patient-reported experience of clinical communication within the secondary care orthodontic setting. The overall mean score per domain is comparable to prior studies (Makoul et al., 2007; Waylen et al., 2015) and a mean percent-excellent score of 88.2% exceeded the expected target of 75% demonstrated in the literature (Catt et al., 2018; Ferranti et al., 2010; Makoul et al., 2007; Waylen et al., 2015). These results are from patient interactions across both treatment and new patient clinics and so should be interpreted within the context of the hospital orthodontic setting. A large proportion of patients are likely to have met their treating clinician on multiple occasions and developed a rapport which may explain the bias towards very positive outcomes. This is reassuring considering the longitudinal nature of orthodontic treatment and the need for an effective clinician–patient relationship to motivate and encourage compliance with our interventions. Reassuringly, the CAT tool has been validated for use in patients who have seen their clinician more than once before (Makoul et al., 2007).

The highest excellent responses were for ‘treated me with respect’ (96%), ‘let me talk without interruptions’ (95.1%) and ‘showed care and concern’ (95.6%). These items are reported to have higher scores in other clinical settings (Ferranti et al., 2010; Makoul et al., 2007). We did not inform clinicians of the session being sampled; however, these results should be viewed in relation to possible Hawthorne effect as clinicians may have had time to read the CAT before patients were seated and hence modified their communication on the day of data collection. Telephone and digital versions of the CAT have been described by Makoul et al. (2007) and this methodology, such as a quick response (QR) code in the reception area, could instead be used in future applications of the CAT.

The lowest scoring item, ‘encouraged me to ask questions’ (55.8%), is the domain most often reported to score lowest on the CAT (Armellino et al., 2021; Catt et al., 2018; Ferranti et al., 2010; Makoul et al., 2007; Waylen et al., 2015). Other lower scoring items included ‘talked in terms I could understand’ (78.9%) and ‘understood my main concerns’ (82.3%). These raise areas of development needed across all clinician groups and are particularly relevant to the shared decision-making process, whereby healthcare decisions are made with the active support of patients (Coulter and Collins, 2011; Da Silva, 2012). A proportion of respondents (12.3%) appeared unsatisfied with their involvement in the decision-making process (item 11). Along with effective communication, these were key themes found to influence orthodontic treatment satisfaction in recent qualitative research (Wong et al., 2018). Without asking the questions and understanding a patient’s main concerns, we cannot invite their true participation in this process (Barber, 2019; Coulter and Collins, 2011; Da Silva, 2012). They are similarly disempowered if we use technical or inaccessible language during clinical interactions. Health professional teams may naturally use more medical terminology when discussing patient care and this may have contributed to some of the responses for ‘talked in terms I could understand’. When discussions happen in the presence of a patient, efforts should be made to clarify their understanding.

The sample size of 204 respondents is in keeping with comparable settings in the existing literature (Catt et al., 2018; Waylen et al., 2015) and we included a relatively large number of clinicians to explore associations between their characteristics and CAT scores, as well as to reflect the workforce of the teaching hospital environment. The use of two centres was also hoped to improve the generalisability of results. Data collection was delayed several times by localised service disruptions and restrictions due to the COVID-19 pandemic, which accounted for the data collection period. Ideally, sampling more clinics repeatedly could have increased the numbers of patient responses for each individual clinician. Patient and clinician sex was recorded as this has been shown to have an influence on communication (Waylen et al., 2015) and both men and women can differ in their communication styles (Hall, 1984). Patient and clinician first language was recorded, as a pilot study on the use of this tool had recognised this as an important confounding factor with respect to patient perceptions of communication (Waylen et al., 2015). The grade of the treating clinician and the region of their primary registrable dental qualification were also noted. There is a diverse workforce within both departments and clinicians may have had different experiences of ‘softer’ skills training  at both undergraduate and postgraduate level. These basic demographics have similarly been collected when using the CAT to assess hospitalist (secondary care) communication skills (Ferranti et al., 2010).

There were no differences between the two centres and no associations were found between clinician characteristics and patient responses on the CAT, except for when English was not the first language of the treating clinician. Here, there were higher odds of achieving an excellent response. This was unexpected but may reflect the large proportion of international postgraduate students in this study who must meet minimum entry requirements in English language proficiency and often undergo undergraduate training in English. It could also be suggested that as non-native speakers, they may use more accessible language when communicating with patients. Similarly, most patients were native English speakers (n=174, 85.3%) and they may have chosen to score clinicians more favourably when recognising English was not their first language. Finding no differences in the percent-excellence responses by clinician sex or grade appears to contradict the findings of a similar study in the dental hospital setting (Waylen et al., 2015) but is in keeping with a larger study into secondary care medical practitioners (Ferranti et al., 2010). We attempted to account for these variables but the inherent positive bias of responses towards ‘excellent’ and the sample being from predominantly female clinicians (65.2%) and postgraduate trainee/registrar grade (62.3%) may have contributed to this. There were also a limited number of responses for the orthodontic therapist grade (n=29, 14.2%) so results may have reduced generalisability for these clinicians.

We have demonstrated that the modified CAT can be implemented in the orthodontic setting and may be useful across other dental specialty and primary dental care services as both an audit tool and patient-reported experience measure. Oversight from the local clinical governance teams meant we did not provide individual feedback reports to clinicians so that the results of this service evaluation could not be used for or against clinicians in the annual appraisal process. This is, however, a beneficial element of the CAT that may be used at the individual or local level to encourage reflective practice around communication and patient–clinician interactions. Any training or development needs in communication identified as a result of this service evaluation are instead intended to be addressed with a team approach within the respective departments. Incorporating patients as key stakeholders in communication skills training has previously been described in the dental education literature (Schönwetter et al., 2012) and the opportunities to raise these findings and enhance existing communication skills workshops is anticipated.

We aimed to gain a snapshot of clinician’s communication skills across the departments but did not examine the effects of clinic type or appointment length on patient responses, although this could be explored in future applications of the CAT in the orthodontic setting. Data on the exact nature of the clinical interaction, such as a consent discussion, could be collected in future studies to elucidate if differences in patient responses exist. Due to the impacts of COVID-19 on clinic templates, there were reduced new patient clinics running at the time of the evaluation and so responses for this clinic type were also relatively small (n=23), limiting the value of subgroup analyses. However, it should be borne in mind that the CAT was specifically designed and tested to be applicable across various settings and to be reflective of the spectrum of clinical interactions that can be held (Makoul et al., 2007). GDC Standards for the Dental Team (2013) are also clear that effective communication skills should be displayed at all phases of patient care. New patient consultations may involve more discussion, but it is at subsequent visits where patients can have the opportunity to ask more questions or discuss aspects of treatment including oral hygiene and appliance wear and care. We could have collected data on whether this was the first time the patient had met their clinician, as in previous studies (Armellino et al., 2021; Waylen et al., 2015) which could have enabled analyses on the effects of clinician–patient rapport on responses. Future applications of the CAT in orthodontics would also benefit from a sample including more new patient consultations.

Another limitation to this study is that it was undertaken in the context of the COVID-19 pandemic. Repeated disruptions to patient care do not appear to have been detrimental to the perceived standards of communication; however, the routine wearing of facemasks, visors and similar personal protective equipment by both patients and clinicians may act as additional barriers to effective non-verbal communication. This reinforces the importance of striving for excellence in the domains of the CAT and ensuring patients have a positive interaction with their clinicians.

## Conclusion

There is a high standard of patient–clinician communication in the hospital orthodontic setting. Key areas of development remain, including encouraging patients to ask questions, talking in terms they can understand, recognising their main concerns and involving them in the decision-making process. The results of this service evaluation can be used to inform communication skills training and be replicated in similar dental settings as part of quality improvement. The CAT only provides a snapshot of a clinical encounter, but there is scope for the results of authentic patient feedback to inform more in-depth qualitative research about the patient experience in orthodontics.
